# Extracorporeal membrane oxygenation for severe COVID-19-associated acute respiratory distress syndrome in Poland: a multicenter cohort study

**DOI:** 10.1186/s13054-022-03959-5

**Published:** 2022-04-07

**Authors:** Ewa Trejnowska, Dominik Drobiński, Piotr Knapik, Marta Wajda-Pokrontka, Konstanty Szułdrzyński, Jakub Staromłyński, Wojciech Nowak, Maciej Urlik, Marek Ochman, Waldemar Goździk, Wojciech Serednicki, Jakub Śmiechowicz, Jakub Brączkowski, Wojciech Bąkowski, Anna Kwinta, Michał O. Zembala, Piotr Suwalski

**Affiliations:** 1 Clinical Department of Cardiac Anesthesia and Intensive Therapy, Medical University of Silesia, Silesian Centre for Heart Diseases, M.Curie-Sklodowskiej 9, 41-800 Zabrze, Poland; 2 Department of Cardiac Surgery, Central Clinical Hospital of the Ministry of Interior and Administration, Warsaw, Poland; 3 Department of Anesthesiology and Intensive Therapy, Central Clinical Hospital of the Ministry of Interior and Administration, Warsaw, Poland; 4 Department of Cardiac Surgery, Central Clinical Hospital of the Ministry of Interior and Administration, Centre of Postgraduate Medical Education, Warsaw, Poland; 5 Department of Cardiac, Vascular and Endovascular Surgery and Transplantology, Medical University of Silesia, Silesian Centre for Heart Diseases, Zabrze, Poland; 6 Department of Anesthesiology and Intensive Therapy, Wroclaw Medical University, Wroclaw, Poland; 7 Department of Anesthesiology and Intensive Care, Jagiellonian University, Medical College, Cracow, Poland; 8 Department of Cardiac Surgery, Heart and Lung Transplantation and Mechanical Circulatory Support, Silesian Center For Heart Diseases, Zabrze, Poland; 9 Pomeranian Medical University in Szczecin, Szczecin, Poland; 10 University of Technology, Katowice, Poland

**Keywords:** COVID-19, ECMO, Intensive care, Mortality, Outcomes

## Abstract

**Background:**

In Poland, the clinical characteristics and outcomes of patients with COVID-19 requiring extracorporeal membrane oxygenation (ECMO) remain unknown. This study aimed to answer these unknowns by analyzing data collected from high-volume ECMO centers willing to participate in this project.

**Methods:**

This retrospective, multicenter cohort study was completed between March 1, 2020, and May 31, 2021 (15 months). Data from all patients treated with ECMO for COVID-19 were analyzed. Pre-ECMO laboratory and treatment data were compared between non-survivors and survivors. Independent predictors for death in the intensive care unit (ICU) were identified.

**Results:**

There were 171 patients admitted to participating centers requiring ECMO for refractory hypoxemia due to COVID-19 during the defined time period. A total of 158 patients (mean age: 46.3 ± 9.8 years) were analyzed, and 13 patients were still requiring ECMO at the end of the observation period. Most patients (88%) were treated after October 1, 2020, 77.8% were transferred to ECMO centers from another facility, and 31% were transferred on extracorporeal life support. The mean duration of ECMO therapy was 18.0 ± 13.5 days. The crude ICU mortality rate was 74.1%. In the group of 41 survivors, 37 patients were successfully weaned from ECMO support and four patients underwent a successful lung transplant. In-hospital death was independently associated with pre-ECMO lactate level (OR 2.10 per 1 mmol/L, p = 0.017) and BMI (OR 1.47 per 5 kg/m^2^, p = 0.050).

**Conclusions:**

The ICU mortality rate among patients requiring ECMO for COVID-19 in Poland was high. In-hospital death was independently associated with increased pre-ECMO lactate levels and BMI.

## Background

The role of extracorporeal membrane oxygenation (ECMO) in the management of severely ill coronavirus disease 2019 (COVID-19) patients continues to evolve. Early data from Wuhan, China, reported an alarmingly high mortality rate of 83% in COVID-19 patients requiring ECMO support [1]. As the pandemic progressed, individual and multicenter reports [2, 3] and early reports from multi-institutional registries [4, 5] presented promising results and significant improvements in survival.

It has been observed, however, that mortality trends for patients with COVID-19-related ARDS supported with ECMO are constantly evolving [6]. Initially, 90-day mortality increased from 36% during the first wave to 48% during the second wave and was no longer comparable to that of non-COVID ECMO-treated patients [7]. Most recently, the unbiased and unselected follow-up claims of the largest German health insurance company [8] clearly indicated that the mortality rate in this group of patients may be even higher than previously reported (exceeding 70%).

At the top of consecutive pandemic waves, Polish health-care system was heavily overloaded. Initially, multidisciplinary hospitals were converted into designated infectious disease centers [9]. Later during the pandemic, when this solution became insufficient, temporary hospitals began to be created. At that time, a large single-center report (and the only report to date on the use of ECMO in COVID-19 patients) was published by Suwalski et al. [10]. The authors reported that between the first and second waves of the pandemic, the mean age of their ECMO patients significantly decreased, while the hospital mortality rate increased from 38 to 68%. These findings were possibly due to novel strains of COVID-19 leading to higher infection rates, a more severe manifestation of the disease, and more COVID-19 hospitalizations and deaths among younger individuals [10]. This observation corroborates with the trends currently being observed in the ELSO database [6].

Presently, the role of ECMO in the management of COVID-related severe respiratory failure remains unclear. Reliable and unbiased data on the outcomes of ECMO treatment for COVID-19 patients from various geographical locations (other than the US and Western Europe) are urgently needed [11]. Our study aimed to shed some light on this issue.

## Methods

The data for this retrospective cohort study was collected from four Polish ECMO centers that were willing to participate in this project. Each participating center had an extensive experience with ECMO before the start of the pandemic and performed at least 20 ECMO procedures in COVID-19 patients during the observation period. Our analysis covered a period of 15 consecutive months from March 10, 2020, until May 31, 2021.

The study included all adult patients with a confirmed COVID-19 infection requiring the use of ECMO during the defined time period. All patients tested positive for COVID-19 and met the stringent inclusion and exclusion criteria issued by the Polish Agency for Health Technology Assessment and Tariff System (AOTMIT) for the implementation of ECMO. These criteria were uniform for all ECMO centers in Poland and included persistent hypoxemia with paO2 / FiO2 < 150 mmHg and / or respiratory acidosis with pH < 7.25 and paCO2 > 60 mmHg, despite conventional ARDS therapy and the use of prone position, protective lung ventilation and muscle relaxants. The exclusion criteria included mechanical ventilation with high peak pressures and/or high oxygen concentration for more than 7 days, irreversible damage to the central nervous system and severe systemic disease with an unfavorable prognosis [12].

Demographic characteristics and details regarding hospital and ECMO treatment, as well as outcomes, were retrospectively analyzed. The Ethics Committee at the Medical University of Silesia in Katowice, Poland, waived the need for informed consent from patients participating in the analysis.

The primary aim of this study was to assess in-hospital mortality of ECMO treatment in the most severe COVID-19 infections and compare the circumstances surrounding ECMO implementation, demographic data, comorbidities, clinical status during ECMO implantation, as well as procedure- and treatment-related variables between non-survivors and survivors during intensive care unit (ICU) stays. The secondary aim was to identify independent risk factors for ICU death.

Depending on the circumstances, ECMO was either established in the ICU of the participating center or the ECMO retrieval team cannulated and transported a patient on ECMO from another facility. Patients were transported either via an ambulance or a helicopter. Mobile ECMO retrieval teams consisting of 2–3 people (usually: an anesthesiologist and/or cardiovascular surgeon and/or perfusionist) were engaged in the transport of an ECMO patient from another facility.

ECMO cannulation was performed percutaneously. For veno-venous (VV) ECMO (representing the vast majority of procedures), the venous drainage cannula was usually inserted in the common femoral vein, while the venous return cannula was usually inserted into the internal jugular vein.

A centrifugal pump integrated with a polymethylpentene hollow fiber oxygenator and heparin-coated tubing was used in all cases. The pump speed was adjusted to obtain an oxygen saturation of greater than 92%. Optimal cannula positioning was verified with a chest X-ray and an ultrasound examination. Intravenous unfractionated heparin was given to maintain the activated partial thromboplastin time at 1.5–2 times normal or to achieve an activated clotting time between 180 and 220 s. The overall goal of invasive mechanical ventilation during VV ECMO was to reduce ventilation invasiveness to achieve the maximal benefit of extracorporeal pulmonary support, based on the ELSO Coronavirus Disease 2019 Interim Guidelines [13].

Comparisons between non-survivors and survivors of their ICU stay were performed. Comparisons between the circumstances surrounding the initiation of ECMO as well as the patient's demographic data, comorbidities, and clinical status at ECMO implantation (Table 1) were conducted. Details on ECMO run, procedure-, and treatment-related variables including the incidence of ECMO complications in both groups (Table 2) were recorded. Definitions of ECMO complications were in accordance with the ELSO Registry [14].

**Table 1:** Circumstances of ICU admission, demographic data, comorbidities, and clinical status at ICU admission to the ECMO center

Group of variables	Variable	All (n = 158)	Death (n = 117)	Survival (n = 41)	p
Circumstances of ICU admission	Admission from another facility	123 (77.8%)	90 (76.9%)	33 (80.5%)	0.799
	Duration of hospital stay before the initiation of ECMO (days)	7.6 ± 6.0	8.1 ± 6.1	6.3 ± 5.7	**0.026**
	Duration of mechanical ventilation before ECMO (days)	5.3 ± 6.2	5.6 ± 6.5	4.8 ± 5.2	0.379
	Transport on ECMO	49 (31.0%)	39 (33.3%)	10 (24.4%)	0.385
	ICU admission after October 1, 2020	139 (88.0%)	107 (91.5%)	32 (78.1%)	0.385
Demographic data	Age (years)	46.3 ± 9.8	47.0 ± 9.6	44.3 ± 10.2	0.164
	Male sex	119 (75.3%)	86 (73.5%)	33 (80.5%)	0.495
	BMI (kg/m^2^)	30.9 ± 5.9	31.4 ± 6.0	29.3 ± 5.0	**0.012**
Comorbidities	Arterial hypertension	41 (25.9%)	33 (28.2%)	8 (19.5%)	0.376
	Chronic pulmonary disease	11 (7.0%)	8 (6.8%)	3 (7.3%)	0.800
	Cancer	4 (2.5%)	4 (3.4%)	0 (0.0%)	0.534
	Psychiatric disorders	7 (4.4%)	6 (5.1%)	1 (2.4%)	0.780
	Thyroid dysfunction	6 (3.8%)	6 (5.1%)	0 (0.0%)	0.316
	Diabetes mellitus	20 (12.7%)	17 (14.5%)	3 (7.3%)	0.356
	Chronic heart failure	3 (1.9%)	3 (2.6%)	0 (0.0%)	0.711
	Coronary artery disease	6 (3.8%)	4 (3.4%)	2 (4.9%)	0.957
	Pregnancy	7 (4.4%)	4 (3.4%)	3 (7.3%)	0.547
	No comorbidities	90 (57.0%)	63 (53.9%)	27 (65.9%)	0.376
Clinical status at ICU admission	paO2/FiO2 ratio (mmHg)	70.5 ± 31.3	68.8 ± 33.4	75.4 ± 24.2	**0.038**
	paCO2 (mmHg)	62.0 ± 22.5	62.3 ± 20.6	61.3 ± 27.5	0.152
	pH (1)	7.33 ± 0.13	7.33 ± 0.12	7.35 ± 0.13	0.201
	Lactate (mmol/L)	1.82 ± 1.12	1.93 ± 1.22	1.51 ± 0.64	**0.008**

Bold indicates significant (p < 0.05)

**Table 2:** Data relating to ECMO treatment

Group of variables	Variable	All (n = 158)	Death (n = 117)	Survival (n = 41)	*p*
ECMO treatment	VV ECMO	156 (98.7%)	115 (98.3%)	41 (100.0%)	0.975
	Extubation on ECMO	7 (4.4%)	2 (1.7%)	5 (12.2%)	**0.018**
	Tracheostomy on ECMO	79 (50.0%)	58 (49.6%)	21 (51.2%)	1.000
ECMO complications	Hemorrhagic	88 (55.7%)	68 (58.1%)	20 (48.8%)	0.394
	Neurological	16 (10.1%)	12 (10.3%)	4 (9.8%)	0.834
	Renal	59 (37.3%)	51 (43.6%)	8 (19.5%)	**0.011**
	Cardiovascular	47 (29.7%)	38 (32.5%)	9 (22.0%)	0.284
	Pulmonary	28 (17.7%)	24 (20.5%)	4 (9.8%)	0.189
	Metabolic	22 (13.9%)	18 (15.4%)	4 (9.8%)	0.526
	Limb ischemia	3 (1.9%)	3 (2.6%)	0 (0.0%)	0.711
	Infection	125 (79.1%)	93 (79.5%)	32 (78.0%)	0.977

Bold indicates significant (p < 0.05)

Data from non-survivors and survivors of their ICU stays were compared. Continuous variables were presented as mean and standard deviation, while categorical variables were presented as percentages. Chi-squared tests, Mann–Whitney U tests, and Student’s t tests were used to test for statistical significance, where appropriate.

Independent variables (listed in Tables 1, 2) were compared between non-survivors and survivors of their ICU stay. The odds ratios for ICU death was calculated for each covariate using a univariable logistic regression model. A progressive stepwise method was then used to obtain a reduced multivariable model. Variables with a *p* value of < 0.05 were then included in the multivariable logistic regression analysis, where a p-value of < 0.05 was considered significant. Based on these calculations, independent risk factors for ICU deaths were identified.

For all analyses, a two-tailed p-value of < 0.05 was considered statistically significant. Analyses and graphs were generated using IBCO Software Inc., (2017). Statistica (data analysis software system), version 13. http://statistica.io.

## Results

Overall, 171 patients were treated with ECMO for refractory hypoxemia due to COVID-19 in participating ECMO centers during the defined time period. In this group, 158 patients (mean age: 46.3 ± 9.8 years, from 21 to 65 years) were analyzed, while 13 patients were excluded because they were still requiring ECMO support at the end of the observation period. Data from all 158 patients were analyzed. In this group, 139 patients (88.0%) were treated during the second and third waves of the COVID-19 pandemic in Poland (after October 1, 2020).

Out of the entire group, 123 patients (77.8%) were transferred to ECMO centers from another facility. The condition of 49 patients (39%) was too severe for safe transport, therefore they were transferred to ECMO centers on extracorporeal life support—41 of these patients (25.9%) were transported with the use of an ambulance, and eight patients (5.1%) were transported using a rescue helicopter. Five patients who were considered candidates for lung transplantation were transferred from one ECMO center to another. (However, this was considered as a single hospitalization for the purpose of statistical analysis.) No deaths or serious complications were recorded during transportation. Among the 158 ECMO implementations, a VV peripheral technique was used in 156 patients (98.7%). The remaining two patients received veno-arterial ECMO due to severe hemodynamic instability.

Circumstances surrounding ECMO implementation, demographic data, comorbidities, and clinical status at ICU admission to ECMO centers are given in Table 1. Longer mean duration of hospital stay before the initiation of ECMO was observed among non-survivors. However, the duration of mechanical ventilation before the initiation of ECMO was similar in both groups.

Patients were predominantly male and the proportion of male patients was similar in non-survivors and survivors. Non-survivors were significantly more obese (Table 1). Overall, 31 patients (19.6%) had BMI values exceeding 35 kg/m^2^. The majority of patients (57%) had no comorbidities before the initiation of ECMO. The mean Charlson Comorbidity Index was similar in both groups (0.9 ± 1.1 vs 0.6 ± 1.0, p = 0.141).

The distribution of comorbidities, present before the start of the ECMO procedure was similar in both non-survivors and survivors. The clinical status analysis is given at the bottom of Table 1. It is clearly seen that there were more patients with profound hypoxemia among non-survivors. Additionally, higher mean lactate values were observed in this group, but the observed range of values was broad (from 0.3 to 11.9 mmol/L).

Data relating directly to ECMO treatment is given in Table 2. Approximately, 50% of patients in both groups received a tracheostomy while on ECMO support. There were significantly fewer patients extubated while on ECMO among the non-survivors.

Complications during ECMO treatment were relatively frequent in the analyzed population. The most common complication was bacterial infections and bleeding (in approximately 80% and 55% of patients, respectively), with similar occurrences among non-survivors and survivors. Another relatively common complication was acute kidney injury requiring renal replacement therapy occurring during ECMO therapy—significantly more frequent in non-survivors, however there were no patients who were dialysis-dependent at the time of the cannulation in our cohort (Table 2).

Among 41 survivors, 37 patients were successfully weaned from ECMO support and were discharged from the ICU in stable condition. Of these, 13 patients (35.1%) were discharged to a different department in the ECMO center and 24 patients (64.9%) were discharged to another facility (usually to the hospital previously referring the patient for ECMO). Four patients underwent successful lung transplantation and were promptly weaned from ECMO after the procedure. They were then discharged home directly from the center where the lung transplant was performed.

Among 117 non-survivors, 113 patients died while on extracorporeal life support. Another four patients died during lung transplantation—either during the procedure (n = 3) or during the postoperative period (n = 1).

The mean duration of ECMO support was 18 ± 13.5 days (from 3 h to 128 days). Seven patients (4.4%) were supported with ECMO for less than 24 h (survival—0%), while 20 patients (12.7%) registered an ECMO run exceeding one month (survival—25%).

The mean duration of ICU stay was 26.3 ± 17.6 days and was significantly shorter in non-survivors (22.6 ± 15.8 vs 36.9 ± 18.4 days, p < 0.001). In the group of survivors, 58.5% of patients stayed in the ICU for more than one month—the corresponding percentage for non-survivors was only 20.5% (p < 0.001).

**Fig. 1:**
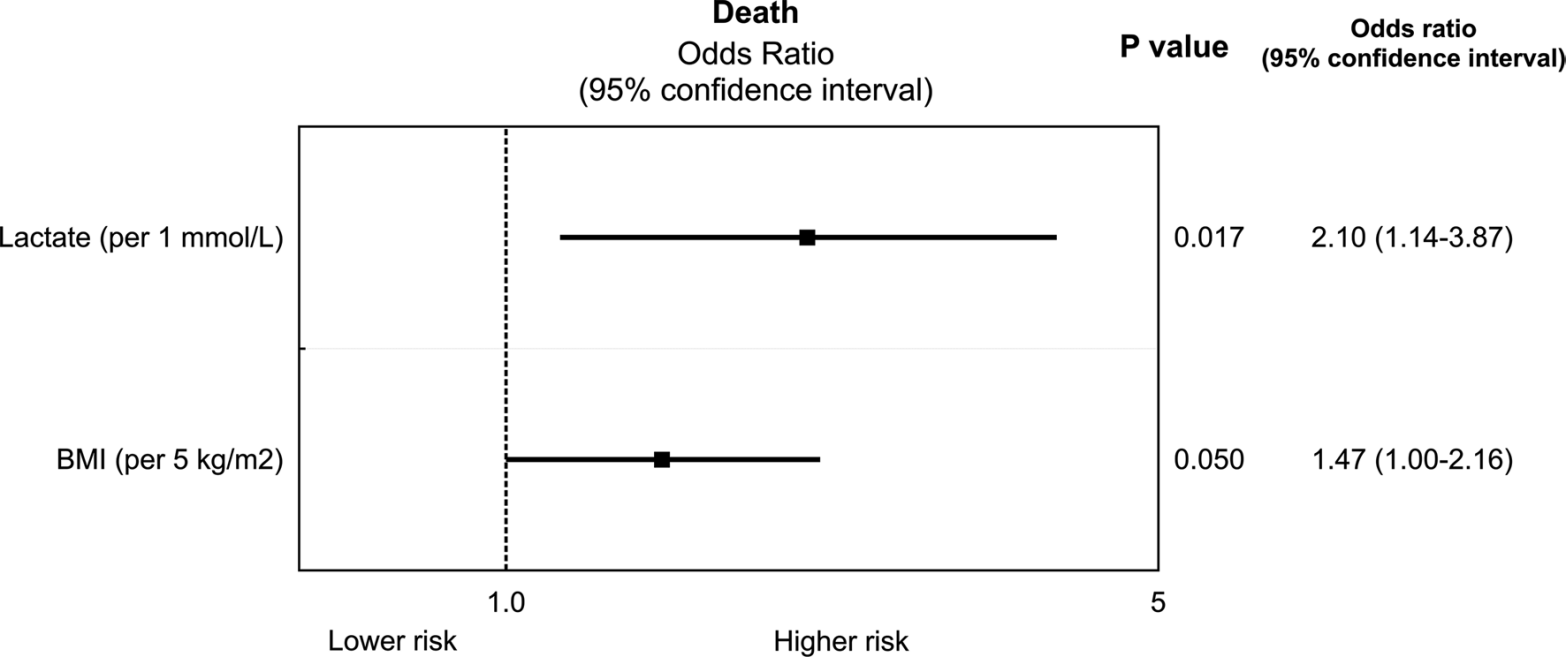
Independent predictors of death in the analyzed population

The crude ICU mortality rate was 74.1%. The results of our multivariable analysis are presented in Fig. 1. Overall, six variables were identified to differentiate non-survivors from survivors in the univariable analysis. In the multivariable analysis, factors that independently influenced ICU death were limited to: pre-ECMO lactate levels (OR: 2.10 per each 1 mmol/L increase, 95% CI (1.14, 3.87), p = 0.017) and BMI (OR: 1.47 per 5 kg/m^2^, 95% CI (1.00, 2.16), p = 0.050). The optimal cutoff point discriminating death and survival was 29.7 kg/m^2^ for BMI (sensitivity 56.4%, specificity 70.7%) and 1.7 mmol/L for lactate levels (sensitivity 60.7%, specificity 65.9%).

**Fig. 2:**
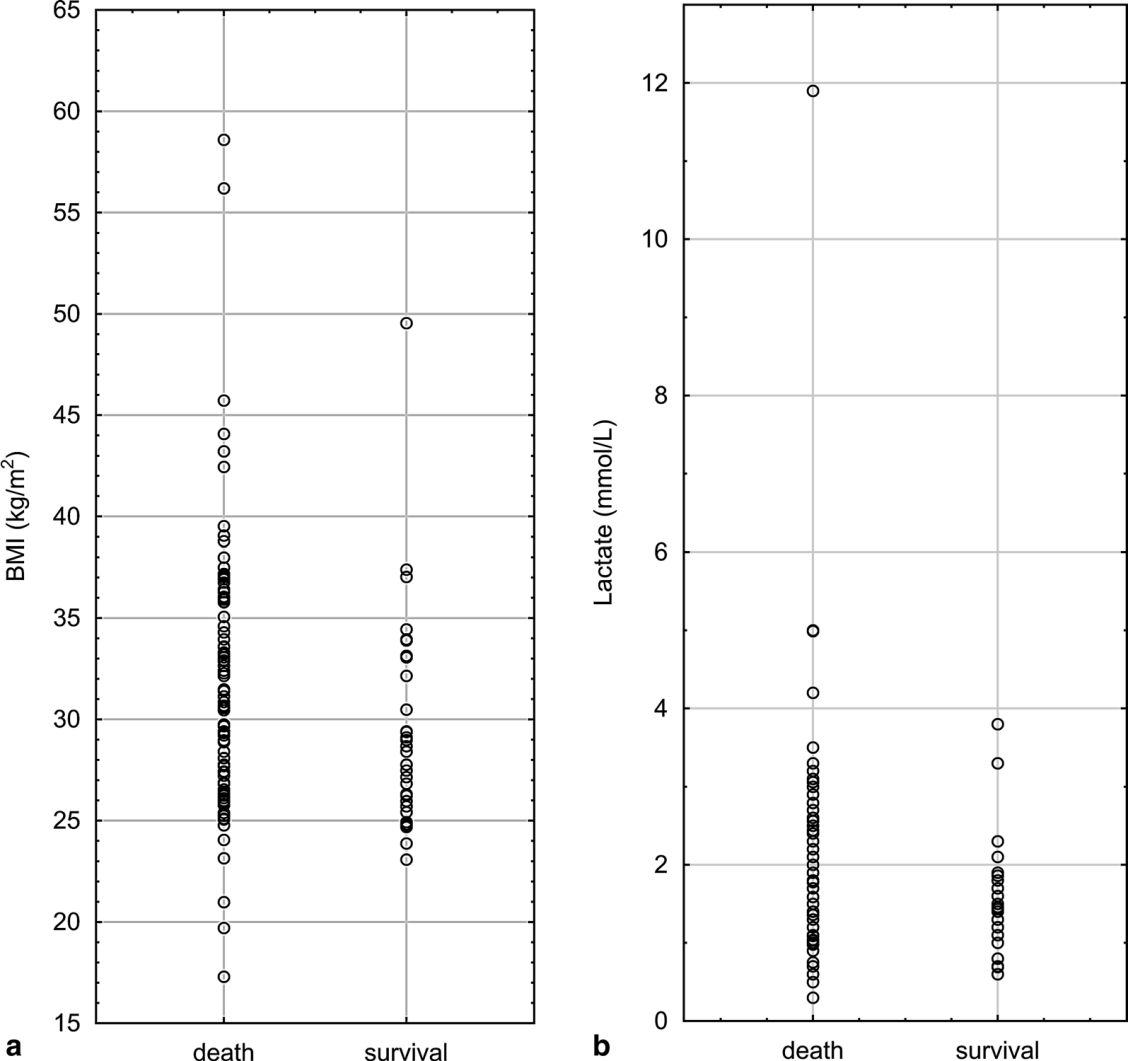
BMI values (left figure) and lactate levels (right figure) at the initiation of ECMO in survivors and non-survivors of ICU stay

In Fig. 2 we presented BMI values (left figure) and lactate levels (right figure) at the initiation of ECMO in survivors and non-survivors of ICU stay.

**Fig. 3:**
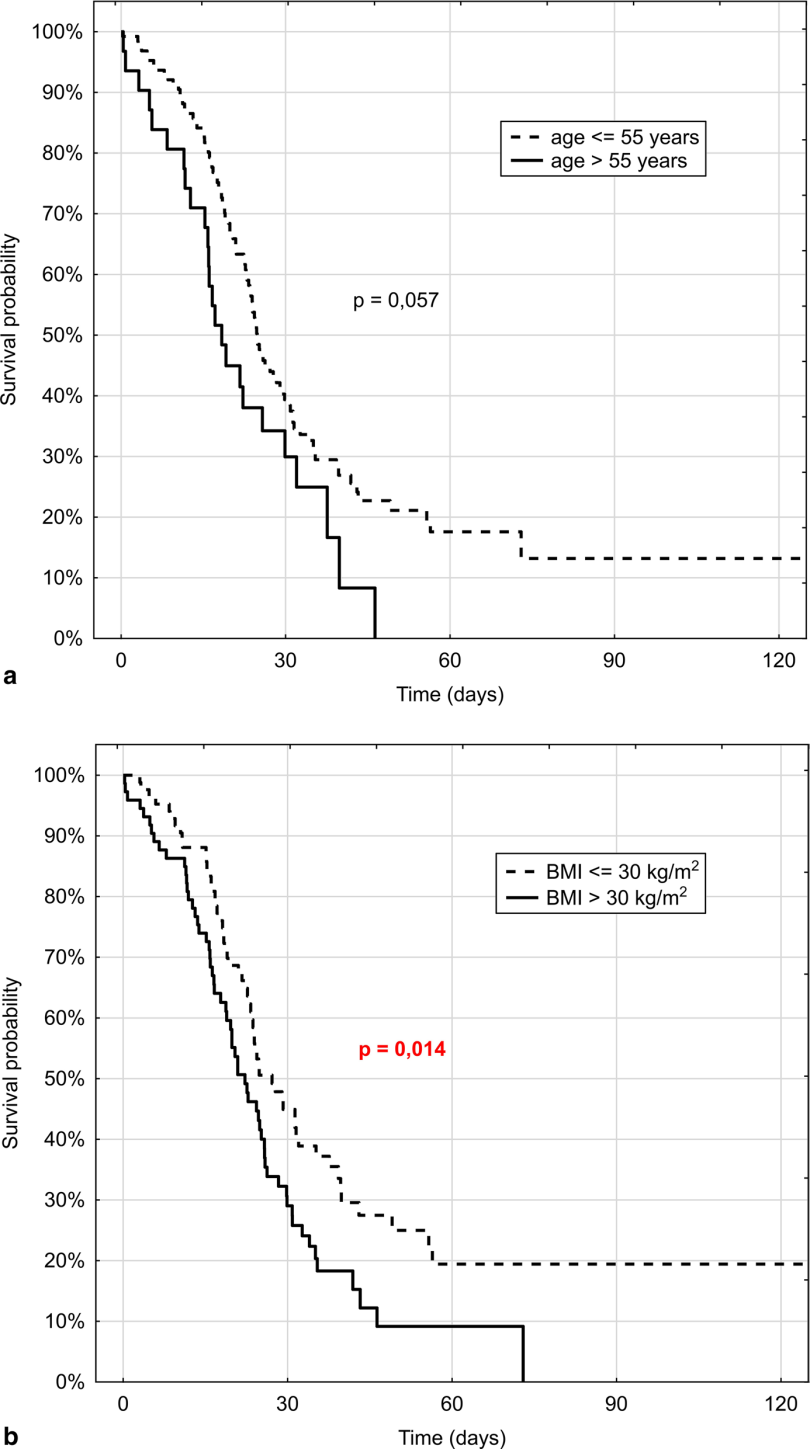
Survival curves for various age groups (> 55 years and ≤ 55 years) (left figure) and obese vs non-obese patients (BMI > 30 kg/m^2^ or ≤ 30 kg/m^2^) (right figure)

In Fig. 3a, b we present survival curves for various age groups (> 55 years and =  < 55 years) and obese vs non-obese patients (BMI > 30 kg/m2 or =  < 30 kg/m2).

## Discussion

In our study, we were able to confirm a high ICU mortality rate among patients requiring ECMO for COVID-19 in Poland. In-hospital death was confirmed to be independently associated with increased pre-ECMO lactate levels and increased BMI.

Our observation period covered the first 15 months of the pandemic in Poland, i.e., the entire first wave, second wave, and a substantial part of the third wave. The severity of the pandemic waves was different in comparison with that observed in other countries. The first wave was relatively benign, due to the prompt adoption of social distancing and a complete lockdown throughout the country [15]. However, Poland was seriously affected by the second and particularly by the third wave of the pandemic [16, 17]

Nearly, a third of the subjects in our study were transported while on ECMO. According to the ELSO Registry—the largest cohort of COVID-19 patients requiring ECMO to date—nearly half of all patients (47%) were transported while on ECMO support [6]. An even greater proportion of COVID-19 patients—approximately 55%—were transported on ECMO support in the Greater Paris region [18]. The difference in utilization of ECMO transport between the international cohort and our study is most likely an indicator of the early stage of development of the ECMO system in Poland.

Current guidelines recommend the application of ECMO in acute respiratory distress syndrome (ARDS) caused by SARS-CoV-2 infection predominantly in patients without significant comorbidities [19]. Interestingly, only 57% of our patients were without comorbidities. Moreover, approximately 20% of patients in our population had a BMI > 35 kg/m2, however—according to the current guidelines—obesity is not considered a contraindication for ECMO in COVID-19 patients [19]. Therefore, it may be hypothesized that VV ECMO—as a relatively infrequent procedure in Poland prior to the current pandemic—was considered a last resort procedure applied in some cases on compassionate rather than medical grounds.

Available data implicates a direct relationship between mortality and the level of hypoxemia at the initiation of ECMO in the COVID-19 population [20]. The very severe pre-ECMO hypoxemia found in our study (with a mean paO2/FiO2 ratio of only 70.5 mmHg) may explain the high mortality rate. Late referral for ECMO and long periods of respiratory support with positive pressure and high oxygen concentration may expose patients to increased risks of ventilation-induced lung injury and patient self-inflicted lung injury.

Unsurprisingly, lactate levels prior to the initiation of ECMO were one of the two independent risk factors for death in our multivariate analysis. This is not surprising, as levels of lactate are related not merely to its production but also its clearance. Therefore, hypoxemia is not the only reason for elevated lactate levels, which may also be a result of impaired oxygen utilization on the cellular level, while acidemia may be observed only in subjects with concomitant renal failure [21].

Veno-venous (VV) ECMO was initially used in a vast majority of cases, in line with the medical literature [2, 3, 6]. However, in selected cases the conversion of VV ECMO to more complex ECMO modes may be needed due to various indications and such a conversion may be required in up to 18% of patients [22].

Approximately, half of our patients received a tracheostomy while on ECMO. There is no consensus on the timing of a tracheostomy in patients on VV ECMO for COVID-19. Hitherto, there is no convincing comparative data concerning this issue, and most of the reports on this subject are case series. The frequency of tracheotomy in this group of patients is also ambiguous and falls within the wide range of 30–61% [8, 23, 24].

There are some negative aspects to performing an early tracheostomy during VV-ECMO use in patients. Bleeding complications are more common, and the hypercoagulable state following discontinuation of anticoagulants raises concerns about the possibility of ECMO circuit malfunction. Due to an increased risk of contaminating personnel, various national organizations recommend caution in this regard [25]. In addition, the technical skills of personnel are decreased by the use of gowns, multiple gloves, and face masks [26].

In our study, only seven patients were extubated on ECMO support. Kunavarapu et al. [27] investigated whether this is a viable treatment option. The authors found that the survival rate was higher in patients placed on ECMO prior to mechanical ventilation, but their sample size for such calculations was relatively small [27].

Complications of ECMO were relatively frequent in the analyzed population. The most common complication was bacterial infections and bleeding (in approximately 80% and 55% of our patients, respectively), occurring with a similar frequency in survivors and non-survivors. Marked immunosuppression (related to the systematic use of corticosteroids), might have contributed to bacterial superinfections, with a predominance of ventilatory-associated pneumonia (VAP). The prevalence of VAP in our study was similar to findings of a recent European study [24] but higher when compared to the data from Latin America [11], and much higher in comparison with the EOLIA trial performed during the pre-COVID era [28].

Another common complication of ECMO in our COVID-19 patients was bleeding. Hemorrhagic complications were reported in 55% of our patients. Similar results were obtained by French investigators, where massive hemorrhages were reported in 42% of their patients [24].

Another relatively common complication was acute renal failure requiring renal replacement therapy. This complication occurred in 37.3% of patients, and was significantly more frequent in non-survivors (43.6% vs 19.5%, p = 0.011). A similar observation was reported in the majority of publications describing complications of ECMO in COVID-19 patients. In two European multicenter cohorts of patients with COVID-19, it was found that 22% and 46% of patients on ECMO required renal replacement therapy [24, 29], while in the largest ELSO registry, renal complications were reported in 42.9% of patients [4]. A systematic review and meta-analysis of twenty-two observational studies including 1,896 patients done by Ramanathan et al. [5] reported that 35% of patients developed renal complications while on ECMO support [5].

The in-hospital mortality in our population was 74.1%. The first experience with ECMO in COVID-19 patients from China revealed very high death rate [1, 30]. Following reports showed significantly lower mortality rates in comparison with our results. In 302 patients from the Greater Paris area, 90-day mortality rate was 54% [18]. Analysis of data from 1,035 US patients from ELSO Registry indicated the mortality of 38% [4], while the European data from the EuroELSO Survey revealed 44% mortality in 1,602 patients [20]. A recent systematic review including 22 studies and 1,896 COVID-19 patients treated with ECMO, found the in-hospital mortality of 37.1% [5].

This striking difference in comparison with our results is difficult to explain. However, a recently published analysis of data from German hospitals (based on data from the German insurance institutions) indicated a mortality rate as high as 71% among 119 patients treated with ECMO during the first wave of COVID-19 pandemic [8]. These results are in line with our findings.

Furthermore, our patients had very late qualification for ECMO treatment based on the extremely low values of the paO2/FiO2 ratio and the long duration of mechanical ventilation before ECMO. Finally, despite available guidelines for ECMO therapy in Poland, a rather low compliance to these guidelines was observed in our study. For example, 14 patients (nearly 10%) were above 60 years old, and 31 patients (approx. 20%) were morbidly obese (BMI > 35 kg/m2).

In our study, we were able to identify two independent risk factors for mortality in ECMO patients—pre-ECMO lactate levels and BMI. The identification of these factors contributing to mortality comes as no surprise.

Prolonged tissue hypoxia seen in patients with COVID-19 plays an important role in the development of subsequent organ dysfunction and mortality. A number of studies have reported the correlation between tissue hypoxia, lactate levels, and mortality among critically ill patients with COVID-19 [31, 32]. We should therefore aim for the early implementation of ECMO before a patient’s lactate level becomes elevated. In some ethically difficult situations, pre-ECMO lactate levels may also help in the decision-making process.

Obesity is a widely discussed risk factor for ICU mortality among COVID-19 patients [33–35]. However, the morbidity and mortality of obese patients managed in ECMO centers remain ambiguous. A recent systematic review including 6 studies and a total of 1,285 patients did not show significant differences between obese and non-obese patients [36]. Ramanathan et al. [5] reported a negative correlation between obesity and mortality in COVID-19 patients treated with ECMO [5]. We should be careful in drawing conclusions from our database. Patients deteriorating on mechanical ventilation (assuming no contraindications for ECMO were identified) should be referred to ECMO centers in a timely manner. According to the most recent ELSO Guidelines, obesity per se is not a contraindication to ECMO [19], although patient selection performed by an experienced clinician must be judicious. In a meta-analysis performed by Zaidi et al., the cutoff value for excessive BMI was set at 30 kg/m^2^, whereas in our study, it was 35 kg/m^2^ [36]. The management of obese patients, especially those with BMIs above 35 kg/m^2^ may be challenging. More data is needed to assess the appropriate utilization of ECMO in patients with high BMIs as this group of patients is under-represented in the literature.

Our study has some significant limitations. This is retrospective research, which is always prone to bias. There were a few high-volume Polish ECMO centers that did not take part in this project. We also have to assume that a significant (but unknown) number of smaller centers also carried out a few ECMO procedures. Therefore, we do not have the entire picture of ECMO utilization for the whole country during the pandemic, as it was possible in a German study that has just been published [37]. Our outcome measures were also limited only to ICU mortality, as we did not have access to the follow-up data of patients successfully discharged from the ICU. We also encountered enormous difficulties in obtaining accurate (or any) data on the circumstances of ECMO implementation—this was particularly true for patients who required ECMO implantation in difficult conditions and remote locations. However, all these deficiencies are balanced by the relatively large sample size which represents a significant part of all ECMO cases performed in Poland during the COVID-19 pandemic.

## Conclusions

Based on our results, it may be concluded that ICU mortality among patients requiring ECMO for COVID-19 was high in Poland. In-hospital death was independently associated with increased pre-ECMO lactate levels and BMI. These results indicate that critically ill, deteriorating COVID-19 patients with severe hypoxia should be referred to ECMO centers in a timely manner. Qualification for ECMO therapy, however, must comply with the current guidelines and have to be performed entirely on medical grounds. This simple reminder comes from an area where there was not much data on ECMO treatment for COVID-19.

## Data Availability

The datasets generated and/or analyzed during the current study are not publicly available due local bioethical regulations in Poland, but are available from the corresponding author on reasonable request.
